# Pesticide Use and Self-Reported Symptoms of Acute Pesticide Poisoning among Aquatic Farmers in Phnom Penh, Cambodia

**DOI:** 10.1155/2011/639814

**Published:** 2010-12-30

**Authors:** Hanne Klith Jensen, Flemming Konradsen, Erik Jørs, Jørgen Holm Petersen, Anders Dalsgaard

**Affiliations:** ^1^Department of International Health, Immunology and Microbiology, Faculty of Health Sciences, University of Copenhagen, 1353 Copenhagen K, Denmark; ^2^Department of Occupational and Environmental Medicine, Odense University Hospital, 5000 Odense C, Denmark; ^3^Department of Biostatistics, Institute of Public Health, University of Copenhagen, 2200 Copenhagen N, Denmark; ^4^Department of Veterinary Disease Biology, Faculty of Life Sciences, University of Copenhagen, 1870 Frederiksberg C, Denmark

## Abstract

Organophosphates and carbamates (OPs/CMs) are known for their acetylcholinesterase inhibiting character. A cross-sectional study of pesticide handling practices and self-perceived symptoms of acute pesticide poisoning was conducted using questionnaire-based interviews with 89 pesticide sprayers in Boeung Cheung Ek (BCE) Lake, Phnom Penh, Cambodia. The study showed that 50% of the pesticides used belonged to WHO class I + II and personal protection among the farmers were inadequate. A majority of the farmers (88%) had experienced symptoms of acute pesticide poisoning, and this was significantly associated with the number of hours spent spraying with OPs/CMs (OR = 1.14, CI 95%: 1.02–1.28). The higher educated farmers reduced their risk of poisoning by 55% for each extra personal protective measure they adapted (OR = 0.45, CI 95%: 0.22–0.91). These findings suggest that improving safe pesticide management practices among the farmers and enforcing the effective banning of the most toxic pesticides will considerably reduce the number of acute pesticide poisoning episodes.

## 1. Introduction

The population of Cambodia is estimated to be more than 14 million [1] with at least 75% engaged in agriculture [2]. Pesticides are not produced in Cambodia but the value of pesticides imported into the country has increased considerably since 1996 [3]. In addition, there is a widespread illegal pesticide trade across the borders [4].

The use of highly toxic pesticides is one of the most significant hazards among agricultural workers in low-income countries and a wide range of acute health effects have been reported [5–8]. In many low-income countries, the safety features highlighted in the Code of Conduct published by the United Nations Food and Agricultural Organization (FAO) [9] are not followed. Studies have found excess use of pesticides, frequent mixing of pesticides, use of substandard equipment, poor personal protection, unsafe storage and disposal of containers, and lack of knowledge on appropriate pesticide management [10–12]. 

A series of studies, mainly from Asia, have documented that the easy availability of pesticides in farming households makes it a preferred means of self-harm. It has been estimated that there are 250,000 deaths annually from pesticide self-poisoning worldwide, accounting for 30% of the suicides globally [13].

Few studies have been conducted in Cambodia on occupational pesticide exposure and associated health risks. A survey conducted by the Environmental Justice Foundation found that inappropriate pesticide use, including its timing, frequency, concentration, and type of pesticides used, are widespread. Safety measures are often ignored or misunderstood and 88% of 210 pesticide sprayers had experienced symptoms of pesticide poisoning [14]. A report from 2004 by CEDAC (Centre d′Etude et de Développement Agricole Cambodgien) found that 33% of pesticides available in the Cambodian market were banned by Cambodian law and that labels were most commonly written in Vietnamese and Thai languages which are incomprehensible to the Cambodian farmers [4]. A small study in Cambodia using qualitative methodologies revealed that untrained sources such as neighbours or pesticide sellers trained farmers in the use of pesticides, there was a lack of appropriate personal protective equipment and that 84% used pesticides which are moderate to extremely hazardous to human health (WHO class Ia, Ib, II ) [15]. However, there is a need to provide more information on pesticide management practices and to determine the health impacts of pesticide use among Cambodian farmers to improve future health interventions. This study therefore aims to describe the types of pesticides used and pesticide handling practices as well as self-perceived symptoms of acute pesticide poisoning among farmers in Boeung Cheung Ek (BCE) Lake, Phnom Penh, Cambodia.

## 2. Methods

### 2.1. Study Area

The investigation was a cross-sectional study carried out in the 3,200 ha wastewater-fed BCE Lake located about 5 km to the north of Phnom Penh, the capital of Cambodia. Household- and industrial wastewater enters the lake untreated and consequently provides nutrients for a widespread culture of water spinach (*Ipomoea aquatica Forsk*.) before the water is discharged into the Bassac River [16]. Water spinach is a perennial aquatic or semiaquatic leafy vegetable of the morning glory family (Convolvulaceae) which is grown in rows secured by a string between two poles to prevent the crops from floating away. The plants are harvested within two to four weeks after seedlings have been transferred and pesticides are regularly applied [17].

### 2.2. Study Design

Data for the study were gathered over a three-month period from August to November 2006 in the villages of Thnout Chrum and Kba Tumnub which are located around BCE Lake. Water spinach cultivation is the main occupation of the farmers living in these two villages [18, 19].

All farmers included in this study had previously participated in a research project by the name PAPUSSA (Production in Aquatic Peri-Urban Systems in South East Asia) [20] and provided the sampling frame for this study. Eighty nine farmers (100% of the active farmers) from the two villages participated in the study and none withdrew during its implementation.

### 2.3. Ethics

After consultation with the village head the farmers signed a written consent form and were informed of their right to refuse participation and to withdraw from the study at any given time. The study was approved by the Ministry of Health in Phnom Penh and the results of the study have been provided to the PAPUSSA project for further dissemination to the farmers.

### 2.4. Questionnaire Survey

A questionnaire-based survey with personal interviews was conducted to assess pesticide handling practices and knowledge, attitudes, and self-perceived health effects of acute pesticide poisoning. The questionnaire was elaborated on the basis of previously applied questionnaires [11]. Under the supervision of the principal investigator the interviews were conducted in the Cambodian language by four students with a bachelor's degree in natural science from the Royal University of Agriculture of Phnom Penh. The questionnaires were later back translated into English. 

Five pilot interviews were carried out with farmers and adjustments to the questionnaire were made accordingly. The questionnaire consisted of a baseline questionnaire which was directed at the household head to capture information about socioeconomic indicators and a monitoring questionnaire used to interview the pesticide sprayers fortnightly to register one-month spraying activity.

### 2.5. Data Analysis

Data were analyzed using the statistical program SPSS (Statistical Products and Service Solution, version 15.0). The electronic entry for each questionnaire was validated against the hard copy questionnaire sheets.

A significance level of 5% was applied in the statistical analyses: *X* ^2^ test, *γ* test, and logistic regression analysis.

### 2.6. Variable Definitions

The outcome variable was defined as “a moderate case of pesticide poisoning” and was defined on the basis of the reported self-perceived symptoms. The symptoms were scored one point for each mild symptom and two points for each moderate symptom they reported. The severity of a self-perceived symptom was assessed with reference to the literature [7]. A total score was defined as the sum of the reported symptoms and dichotomized below the median.

### 2.7. Statistical Analysis

In the marginal analyses all exposures and confounders were tested against the outcome “a moderate case of pesticide poisoning” using *X* ^2^ test for coherence and *γ* test for strength of coherence. Potential confounders were age, sex, body mass index (BMI), and socioeconomic indicators (house size, monthly pesticide expenditure, total size of cultivated land, family members working outside the household, and highest educational level within the household).

In the logistic regression analysis, the effects of exposures were investigated while taking into account possible confounders. The first exposure variables included in the model as continuous variables were pesticide spraying frequency and number of hours spent spraying with OPs/CMs the preceding month. The effect of the farmers' protective measures was then estimated both as a categorical variable and as an aggregated continuous variable created by assigning one point to each protective measure they had adapted.

Additionally, socioeconomic indicators were tested as aggregated variables by grouping the farmers into high, middle and low socioeconomic status. This was done by assigning points from one to three based on the 95% confidence interval (one point: <95% CI, two points: = 95% CI, three points: >95% CI) of the numerical variables. The variable “highest educational level within the household” was assigned the following scores. One point: no education, primary school; two points: lower secondary, and three points: upper secondary/technical/university. The aggregated variable was then categorized into three categories based on the percentiles.

## 3. Results

### 3.1. Description of Participants

The farmers were recruited from 93 households with a total of 113 farmers of which 89 were pesticide sprayers. The pesticide sprayers were mainly men (70%) with an average age of 38 years (range 17–69). The farmers were all Khmer and practiced Buddhism.

### 3.2. Pesticides Used

The pesticides used by the farmers in BCE Lake are presented in Table 1 and listed according to their WHO classification. Insecticides, mainly the highly hazardous organophosphates (class Ia/Ib) which are banned or restricted in use, and the moderately hazardous pyrethroids (class II), were commonly used by the farmers. As many as 50% of the pesticides used belonged to WHO class I + II followed by class III (19%), obsolete (6%), and unclassified (25%). 

### 3.3. Pesticide Handling

The mean years of working with pesticides were nine years (range 1–25). The farmers generally perceived pesticides as a crucial necessity for growing water spinach. Some even stated that it would be impossible to grow a good crop without them. 

The knowledge and attitudes among the farmers towards pesticides are shown in Table 2. A vast majority (91%) believed pesticides enter their body and also that they have a deteriorating effect on their health, 46% of the farmers claimed that they followed the instructions set out on the labels on the pesticide containers, and 69% had received instruction on the use of pesticides mainly from salesmen and neighbours. 

All the farmers routinely mixed between four and six pesticides in one spray producing a “chemical cocktail”. During the interviews, a majority of the farmers stated that mixing multiple types of pesticides made the pesticides stronger and more effective and some said that the pesticides do not work efficiently when sprayed one at a time. 

The level of personal protection and hygiene measures are shown in Table 3. Half of the farmers claimed to use a mask when applying pesticides, 18% used gloves, and 3% used boots. 

Pesticides were commonly stored inside their house; 15% stated that they kept them in their house (kitchen, living room, or bedroom) and 28% reported that they stored them in their house beyond reach of children and animals. Only one farmer claimed to use a padlock for the safe storage of pesticides. The remaining 55% households reported outside storage (14% under a shelter, 30% under the house, 8% in a tree, and 3% in the lake).

### 3.4. Symptoms and Risk Factors

Table 4 shows the prevalence of self-reported symptoms according to their manifestation and severity. The majority of the pesticide sprayers (88%) had experienced symptoms of acute pesticide poisoning in relation to spraying activities the preceding month. The most common moderate symptoms were blurred vision, muscle cramps, chest pains, excessive sweating, body tremors and shortness of breath. Among the most common mild symptoms were dry throat, dizziness, headache, fatigue, joint pains, itchy skin, muscle weakness, and nausea. 

**Figure 1:**
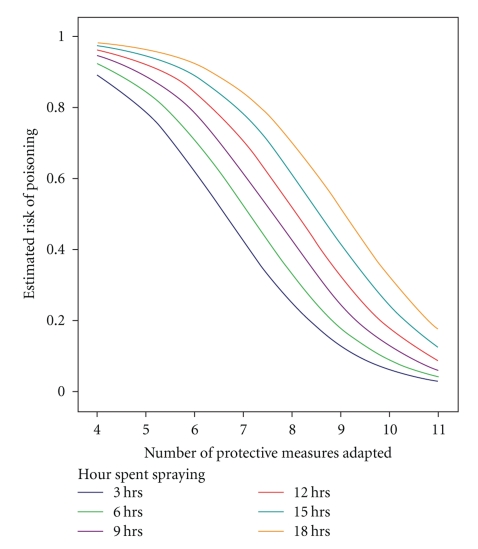
Estimated risk of acute pesticide poisoning for farmers of high educational level.

The final logistic regression model (Table 5) found that for each extra hour a farmer spent spraying with OPs/CMs the risk of having experienced a moderate case of pesticide poisoning increased by 14% (OR = 1.14, CI 95%: 1.02–1.28). This increase was statistically significant (*P* = .002). The model suggested an interaction between the educational level and the number of protective measures adapted (*P* = .051). The high educated farmers reduced their risk of experiencing a moderate case of pesticide poisoning by 55% (OR = 0.45, CI 95%: 0.22–0.91) for each extra protective measure they adapted (Figure 1), while the low educated farmers showed no significant risk cut-back. 

The figure represents farmers with a high educational level in Boeung Cheung Ek Lake (*P* = .026) and shows the predicted probabilities for experiencing acute pesticide poisoning at different protective levels and the number of hours spent spraying with Ops/CMs. For example, the farmers who adapted seven protective measures and sprayed 12 hours had a predicted probability of 0.74.

## 4. Discussion

This study found a frequent use of the most toxic insecticides belonging to the WHO classification Ia + Ib which are, respectively, extremely and highly hazardous (41%) and class II which are moderately hazardous (18%). Both classes are banned or have restricted use in Cambodia, and the widespread use indicates a limited capacity by the authorities to enforce directives regulating pesticide use [4]. 

These findings are in accordance with studies from other low-income countries where highly acute toxic pesticides are frequently used [21, 22]. This is probably due to the perception among small-holder farmers that the broad spectrum class I + II pesticides are more effective and easy to use. The farmers often lack the knowledge to correctly identify the pests attacking their crops and therefore fail to select a narrow-spectrum pesticide [23]. Another plausible reason might be the fact that the most toxic pesticides, often banned or restricted in use, are the cheapest on the market [22].

The presence of banned pesticides in Cambodia seems mainly due to illegal import particularly from Thailand and Vietnam by private companies, traders, and vendors. Such illegal imports demand political attention and increased effort by the Cambodian Government to regulate the private sectors. This is especially important given the general inability of poor farmers in Cambodia and other low-income countries to ensure safe use of pesticides. Similar widespread illegal imports, sales, and use of banned pesticides are also common in Latin American and African countries [11, 22, 24, 25] calling for an increased global attention to the issues. 

Pesticide containers were all labelled in either Vietnamese or Thai, languages which are incomprehensible to the farmers in BCE Lake. However, 46% of the farmers stated that they read and followed the label instructions. This statement indicates that the question has been misinterpreted by the farmers who perceive the information they received from family members, neighbours, or salesmen as in agreement with the label instructions. According to a subdecree issued by the prime minister of Cambodia in 2001 and the Stockholm Convention [4] signed by the Cambodian Government, label instructions written in Khmer are mandatory. This indicates a lack of enforcement power by the Cambodian Government or unwillingness to enforce current laws [26].

Pesticides were most commonly stored inside the house within easy reach of children and close to food commodities resulting in a potential great risk of daily unintentional exposure. Also, the easy availability of highly toxic pesticides makes household members vulnerable to self-harm attempts and suicides [27]. Banning severely toxic pesticides have been successful in reducing deaths from suicides in other low-income countries such as Sri Lanka [28], and farmers in Cambodia should be encouraged and supported to improve the storage of pesticides reducing poisoning of food products, accidental poisoning, and self-poisoning episodes. 

Despite the fact that the farmers had some awareness of the health hazards associated with pesticide use, they did not protect themselves adequately from acute pesticide poisoning. All farmers wore inadequate personal protective equipment leading to unsafe protection when mixing and spraying pesticides. A high percentage said they had received instructions either at the market or from a neighbour but the dilemma is that there is no legislative control requiring pesticide users and salesmen to be formally trained in safe work practices. 

Evaluation of personal protective equipment showed that roughly half of the farmers (44%) used a mask when mixing and spraying pesticides. From field observations, it was noted that these masks were disposable cotton masks not manufactured for pesticide spraying and the level of protection from such masks is unknown. Items like gloves, boots, and face protective screen were rarely used, especially considering the high awareness among the pesticide sprayers about exposure through the skin. The reasons for the limited use of personal protective equipment varied. Some stated that it hindered their ability to work; others said it was uncomfortable to wear protective equipment in the humid climate and it was difficult to breathe properly through a mask. Similar findings are seen in other studies where the use of personal protective equipment or the contrary was seen to depend on having experienced pesticide-related health problems or not, age, pesticide application frequencies, and the perception of personal protective equipment being uncomfortable in the hot climate or hindered work [29, 30].

With regard to the protective behaviour adapted during and after spraying operations, the study found that a large proportion of the farmers were presumably highly protected according to the answers they provided. The pesticide sprayers rarely drank, smoked, and ate while spraying and hygiene measures such as changing clothes, washing hands, and showering after spraying pesticides were common practice. This finding contrasts to a study conducted in Bolivia by Jørs et al., who documented a low percentage of farmers taking appropriate protective measures. This difference might be due to the availability of water and thus reflect the hygienic behaviour of the general population [11]. 

Our study demonstrates that the number of hours spent spraying with OPs/CMs was a statistically significant risk factor for the farmers' risk of having experienced a moderate case of pesticide poisoning. Furthermore, our study showed that the farmers with a high level of education (upper secondary, technical and university) had a reduced risk for each extra protective measure they adapted. This has also been seen in other studies, where the use of personal protective equipment and personal hygiene measures reduced the risk of poisoning [10, 11, 31]. In comparison, the low educated farmers (no education, primary school, and lower secondary school) had no significant risk reduction. Interestingly, there was no indication of the lower educated taking fewer precautions or using more OPs/CMs than the high educated farmers. One explanation for this striking difference could be that the low educated farmers have a lower ability to link their symptoms to the use of pesticides. Another explanation could be that the quality of the personal protective equipment used is unknown but the possible diverging qualities might influence the effectiveness of the individual protective level. Whether the interaction between educational level and the number of protective measures adapted reflects the reality among farmers in BCE Lake can be discussed due to the scarce borderline significance (*P* = .051). However, the interaction does not seem entirely unreasonable from a chain of logic point of view. 

Recall bias of symptoms may have occurred due to the possible inability of farmers to recall symptoms in connection with each spraying session two weeks in retrospect. It must be presumed though that the more serious the symptoms are the easier the recall is. 

The interviewers as well as the respondents were aware of the nature of the study, and some of the positive relationships between exposure and the degree of poisoning may have been due to bias in reporting. 

The study population was a purposive sample and not randomly selected from the total population which limits the study to conclusions on the situation in BCE Lake. The study may not fully represent the farmers' perception of pesticide poisoning given the nature of the unique farming system under study. However, the findings compare with the symptoms and the pesticide handling practices found in previous studies in Cambodia from vegetable growing areas in Kandal, Siem Reap, and areas around Phnom Penh [15].

The study could have been strengthened by including a control group of farmers not spraying pesticides. However, this was not possible since the culture of water spinach requires application of pesticides and a control group involved in this type of production could therefore not be identified.

## 5. Conclusion

From this study it was evident that symptoms of occupational pesticide poisoning were common among farmers and were related to the number of hours spraying OPs/CMs per month and the number of personal protective measures adapted. Highly toxic pesticides belonging to WHO class I + II (59%) and banned or restricted by the Cambodian law were widely used. Although the farmers had some awareness of the dangers of pesticides, they did not protect themselves adequately from acute pesticide poisoning. A first priority must be to effectively phase out the most hazardous pesticides from the market, control pesticide imports and sales, and educate farmers in the proper use of pesticides.

## Figures and Tables

**Table 1:** Pesticides used by 93 households in Boeung Cheung Ek Lake, Phnom Penh, Cambodia.

Main use	Chemical type	Active ingredient	Reported use %	WHO Classification^(a)^
Insecticides	Organophosphates	Monocrotophos	11.8	Ib
		Dichlorvos	71.9	Ib
		Mevinphos	11.8	Ia
		Methidathion	1.1	Ib
		Methamidophos	5.4	Ib
	Pyrazole	Fipronil	1.1	II
	Thiazole	Thiamethoxam	1.1	III
	Neonicotinoid	Imidacloprid	3.2	II
	Pyrethroids	Cypermethrin	93.5	II
	Organochlorine	Endrin	2.2	O

Fungicides	Dithiocarbamates	Mancozeb	44.1	U
		Zineb	48.4	U
		Propineb	64.5	U
	Benzimidazoles	Carbendazim	2.2	U
	Hydroxides	Copper hydroxide	73.1	III
		Copper oxychloride	46.2	III

^(a)^Ia: extremely hazardous, Ib: highly hazardous, II: moderately hazardous, III: slightly hazardous; U: unlikely to present acute hazard in normal use, O: obsolete.

**Table 2:** Knowledge and attitudes towards pesticides among 113 farmers in Boeung Cheung Ek Lake, Phnom Penh, Cambodia.

	Prevalence %	Fraction
Believe pesticides have a deteriorating effect on their health condition	90.9	100/110
Believe pesticides can enter the body	89.4	101/113
Routes of exposure:		
Dermal	84.0	89/106
Inhalation	42.5	45/106
Oral	25.5	27/106
Using pesticides for other purposes than the intended use	2.2	2/93
Throwing empty pesticide bottles in the lake	100.0	92/92
Keeping pesticide safely locked up	1.1	1/92
Follow label instructions	45.7	42/92
Receiving instructions from:	68.8	64/93
Salesman	65.6	42/64
Neighbour	51.6	33/64
Family member	6.3	4/64
Course	1.6	1/64
Giving correct interpretation to the following pictograms:		
Toxic compound	59.3	54/112
Keep pesticides safely locked up	29.5	33/112
Wear protective clothing	62.8	71/112
Wear boots	83.9	94/112
Wear screen	70.8	80/113
Wear gloves	97.3	109/112
Wear mask	95.6	108/113
Dilute pesticide with water	53.1	60/113
Use pump pointing it to the ground	81.4	92/113
Wash hands after spraying	70.3	78/111
Environmental hazard	41.6	47/113

**Table 3:** Personal protective measures adapted by 89 farmers in relation to spraying pesticides in Boeung Cheung EK Lake, Phnom Penh, Cambodia.

	Prevalence %	*N*
Clothing		
Long-sleeved shirt	85.4	76
Trousers	86.5	77
Hat	93.3	83
Personal protective equipment		
Mask	49.4	44
Gloves	18.0	16
Boots	3.4	3
Screen	1.1	1
Hygienic measures		
No eating while spraying	87.6	78
No smoking while spraying	91.0	81
No drinking while spraying	80.9	72
Re-entry time > 48 hrs.	35.6	31
Changing clothes after spraying	95.5	85
Washing hands after spraying	100.0	89
Taking a shower after spraying	97.8	87
Not sucking the nozzle when obstructed	97.8	87

**Table 4:** Reported self-perceived symptoms among 89 farmers in Boeung Cheung Ek Lake, Phnom Penh, Cambodia.

Types of symptom	*N *	Prevalence	Severity of symptoms:	
		%	Mild	Moderate
Muscarinic symptoms				
Headache	49	55.1	+	
Blurred vision	23	25.8		+
Chest pain	14	15.7		+
Excessive sweating	13	14.6		+
Shortness of breath	11	12.4		+
Nausea	9	10.1	+	
Excessive salivation	6	6.7		+
Stomach ache/cramp	5	5.6		+
Cough	5	5.6	+	
Vomiting	3	3.4		+
Diarrhea	2	2.2		+
Nicotinic symptoms				
Muscle cramp	20	22.5		+
Muscle weakness	13	14.6	+	
Twitching eyelids	3	3.4		+
CNS symptoms				
Dizziness	51	57.3	+	
Fatigue	41	46.1	+	
Body tremor	9	10.1		+
Numbness	10	11.2		+
Insomnia	3	3.4	+	
General signs				
Dry throat	61	68.5	+	
Joint pain	34	38.2	+	
Itchy skin	22	24.7	+	
Red eyes	7	7.9	+	
Cold limbs at night	6	6.7	+	
Burning nose	4	4.5	+	
Loss of appetite	1	1.1	+	
Runny nose	2	2.2	+	

**Table 5:** Logistic regression model for the risk of acute pesticide poisoning among 87 farmers in Boeung Cheung Ek Lake, Phnom Penh, Cambodia.

	OR	95% CI	*P*
Number of hours spent spraying with organophosphates and carbamates	1.141	1.02–1.28	.002
Risk reduction pr. number of protective measure adapted for farmers with a high educational level^(1)^	0.446	0.22–0.91	.026
Risk reduction pr. number of protective measure adapted for farmers with a low educational level^(2)^	1.054	0.66–1.70	.828

^(1)^High educational level: upper secondary, technical school, and University. ^(2)^Low educational level: no education and primary school.
